# Segmental Duplications Arise from Pol32-Dependent Repair of Broken Forks through Two Alternative Replication-Based Mechanisms

**DOI:** 10.1371/journal.pgen.1000175

**Published:** 2008-09-05

**Authors:** Celia Payen, Romain Koszul, Bernard Dujon, Gilles Fischer

**Affiliations:** Institut Pasteur, Unité de Génétique Moléculaire des Levures, CNRS, URA2171, Université Pierre et Marie Curie-Paris 6, UFR927, Paris, France; Brandeis University, United States of America

## Abstract

The propensity of segmental duplications (SDs) to promote genomic instability is of increasing interest since their involvement in numerous human genomic diseases and cancers was revealed. However, the mechanism(s) responsible for their appearance remain mostly speculative. Here, we show that in budding yeast, replication accidents, which are most likely transformed into broken forks, play a causal role in the formation of SDs. The Pol32 subunit of the major replicative polymerase Polδ is required for all SD formation, demonstrating that SDs result from untimely DNA synthesis rather than from unequal crossing-over. Although Pol32 is known to be required for classical (Rad52-dependant) break-induced replication, only half of the SDs can be attributed to this mechanism. The remaining SDs are generated through a Rad52-independent mechanism of template switching between microsatellites or microhomologous sequences. This new mechanism, named microhomology/microsatellite-induced replication (MMIR), differs from all known DNA double-strand break repair pathways, as MMIR-mediated duplications still occur in the combined absence of homologous recombination, microhomology-mediated, and nonhomologous end joining machineries. The interplay between these two replication-based pathways explains important features of higher eukaryotic genomes, such as the strong, but not strict, association between SDs and transposable elements, as well as the frequent formation of oncogenic fusion genes generating protein innovations at SD junctions.

## Author Summary

Duplications of long segments of chromosomes are frequently observed in multicellular organisms (∼5% of our genome, for instance). They appear as a fundamental trait of the recent genome evolution in great apes and are often associated with chromosomal instability, capable of increasing genetic polymorphism among individuals, but also having dramatic consequences as a source of diseases and cancer. Despite their importance, the molecular mechanisms of formation of segmental duplications remain unclear. Using a specifically designed experimental system in the baker's yeast *Saccharomyces cerevisiae*, hundreds of naturally occurring segmental duplications encompassing dozens of genes were selected. With the help of modern molecular methods coupled to detailed genetic analysis, we show that such duplication events are frequent and result from untimely DNA synthesis accidents produced by two distinct molecular mechanisms: the well-known break-induced replication and a novel mechanism of template switching between low-complexity or microhomologous sequences. These two mechanisms, rather than unequal recombination events, contribute in comparable proportions to duplication formation, the latter being prone to create novel gene fusions at chromosomal junctions. The mechanisms identified in yeast could explain the origin of a variety of genetic diseases in human, such as hemophilia A, Pelizaeus-Merzbacher disease, or some neurological disorders.

## Introduction

In humans, segmental duplications (SD) cover up to 5.2% of the genome [1] and are responsible for numerous gene-dosage imbalances [2], gene fusions and disruption events 3,4,5. Together with large insertions/deletions, SDs lead to gene copy number variations (CNVs) which represent a major source of polymorphism between individuals [6]. They have been associated with the development and evolution of both cancers [7],[8],[9],[10],[11] and genetically complex phenotypes such as predisposition to autism [12], epilepsy [13], Alzheimer disease [14], glomerulonephritis [15], systemic autoimmunity [16] and susceptibility to HIV/AIDS infections [17]. A specific mapping of CNVs on human chromosome 22 revealed that more than 2/3 of the breakpoints intersect with SDs [18]. This strong correlation reflects the similar nature of CNVs and SDs and suggests tightly coupled co-evolution mechanisms [19].

**Figure 1:**
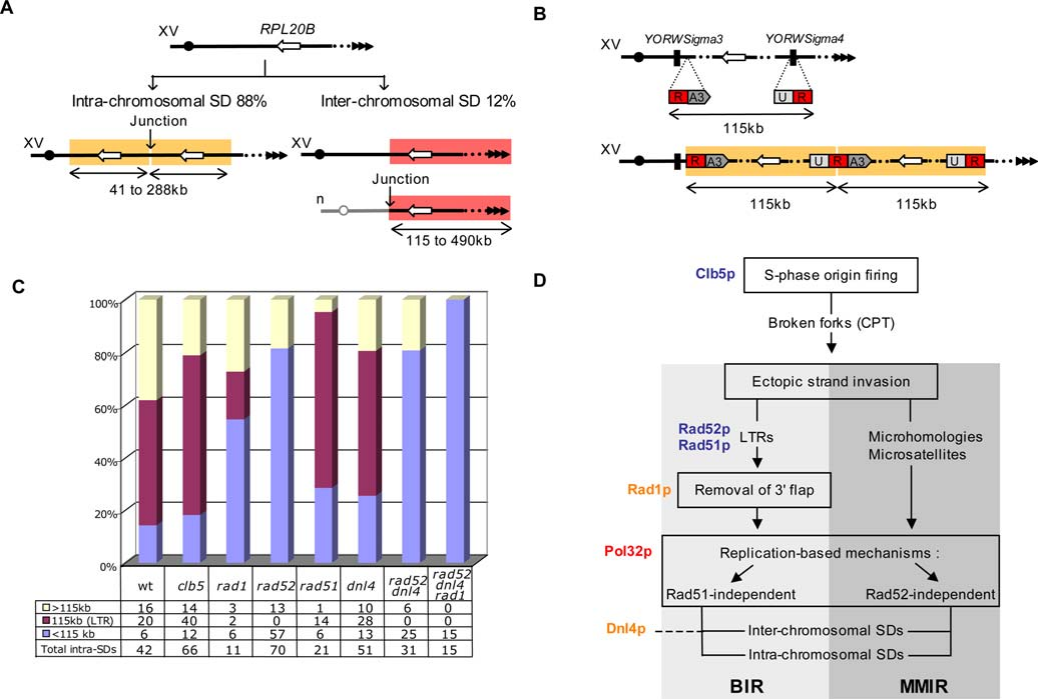
Segmental duplication assays. (A) Growth recovery assay [20]. Black circles and triangles represent centromeres and telomeres, respectively. White open arrow represents the *RPL20B* gene (YOR312C) whose duplication is selected for. Yellow and pink boxes denote intra- (left) and one type of inter-chromosomal (right) duplications, respectively. A non-reciprocal translocation event between the right arm of chromosome XV and another chromosome (denoted “n”) is represented: for other types of inter-chromosomal SD (i.e. chimerical supernumerary chromosome and unequal reciprocal translocation, see [20] and [21]). SD size ranges are indicated below the double-headed arrows. (B) Uracil prototrophy recovery assay. Top: schematic representation of the right arm of chromosome XV spanning the *RPL20B* locus and the two flanking Ty3 LTRs (*YORWsigma3* and *YORWsigma4*) located 115 kb apart from each others. 5′- and 3′-truncated are either inserted next (*YORWsigma3*) or replaces (*YORWsigma4*) Ty3 sequences. The “R”-labeled red box indicates the 58 or 401 bp overlap between the two truncated URA3 cassettes. Bottom: a functional *URA3* gene restoring uracil prototrophy is generated through 115 kb direct-tandem duplication events involving the overlapping sequences. (C) Size distribution of intra-chromosomal SDs. The x and y-axis of the diagram indicate the strain background and the percentage of events recovered, respectively. Yellow, violet and blue bars represent the proportion of duplications larger, equal to and smaller than 115 kb, respectively (with the actual number of events analyzed indicated in the table below). (D) Phenomenology of SD formation. Protein names involved in the different steps are indicated to the left of the diagram. Red, orange and blue names represent proteins whose deletions abolish, reduce and increase SD formation, respectively. Light and medium grey boxes indicate the two alternative mechanisms of SD formation, BIR (Break-induced Replication) and MMIR (Microhomology/Microsatellite-induced Replication), respectively. CPT = camptothecin.

We previously designed a gene dosage assay in *Saccharomyces cerevisiae* to screen for the spontaneous duplication of a single gene, *RPL20B* [20]. Although the size of this gene is relatively small (1.6 kb), no single gene duplication was ever found. Instead, only intra- and inter-chromosomal duplications of large DNA segments, encompassing dozens of neighboring genes, were recovered (88% and 12%, respectively, Figure 1A) [20]. These findings showed that spontaneous SDs can compensate for gene dosage imbalance by altering gene copy number in the yeast genome and that CNVs can encompass numerous genes. Approximately half of the SD junctions involved dispersed repeats such as Long Terminal Repeats (LTRs) from Ty retroposons, while the other half consisted of low complexity DNA sequences (poly A/T, trinucleotide repeats), as well as microhomologous sequences whose identity spans only over a few nucleotides in length. The location and the type of sequences found at the breakpoints suggested that SDs might result from replication accidents improperly repaired through both homologous and non-homologous recombination events [20]. In order to explore the mechanisms of SD formation, we deciphered how perturbations of the replication process and of double strand break (DSB) repair pathways affect rates, types, sizes and breakpoint sequences of duplications. Providing the largest set of experimentally generated *de novo* duplications, the present study describes 338 independent SDs recovered in different mutant backgrounds and culture conditions. We show that replication-generated DNA ends are converted into large SDs through both homology-dependent and -independent replication-based mechanisms.

## Results

### High Rate of Spontaneous SD Formation

Two highly similar paralogous genes, *RPL20A* (YMR242c) and *RPL20B* (YOR312c), encode the Rpl20 yeast ribosomal protein. The deletion of *RPL20A* results in a marked slow-growing phenotype which can be compensated by the spontaneous duplication of *RPL20B* [20]. Slow growing parental strains (*rpl20AΔ*) are propagated through serial transfer into rich medium. Rapidly growing revertants among slow growing populations are isolated by regularly plating aliquots of the cultures at each transfer step. Using this assay, we re-estimated the spontaneous duplication rate of *RPL20B* to be 1×10^−7^ SD/cell/division (Luria-Delbruck fluctuation tests using the 0 term of the Poisson law (p = 1−e^lnf0/ndiv^; see Methods; Table 1). This value is higher than previously estimated (between 2×10^−9^ and 10^−10^ SD/cell/division [20],[21]) due to an initial underestimation of the time needed for a duplication-carrying cell to overtake the population of the slow growing parental cells (see Methods).

**Table 1:** SD characteristics in different genetic backgrounds.

SD assay	relevant genotype	duplication rate	fold change/WT	number of SDs analyzed^b^		LTR-mediated SDs	
				intrachromosomal	interchromosomal	%	p-values
growth recovery (*rpl20AΔ*)	WT	1.0×10^−7^	**1**	42 (20)^a^	6 (3)^a^	48	-
	*clb5*	7.3×10^−5^	730	66 (40)	3 (3)	62	0.046
	WT + CPT (10 µg/ml)	3.2×10^−5^	320	22 (12)	0	54	0.121
	WT + HU (100 mM)	2.1×10^−7^	2.1	16 (2)	3	11	0.003
	*mrc1^AQ^*	5.3×10^−7^	5.3	5 (1)	0	20	0.199
	*mrc1^AQ^ +* HU (100 mM)	4.4×10^−7^	4.4	10 (1)	0	10	0.024
	*pol32*	<6.9×10^−9^	<0.07	0	0	0	-
	*rad1*	2.1×10^−8^	0.2	11 (2)	3 (nd)	14	0.056
	*rad52*	2.8×10^−−7^	2.8	70 (0)	1 (0)	0	<10^−6^
	*rad51*	7.7×10^−7^	7.7	21 (14)	10 (8)	71	0.025
	*dnl4*	8.3×10^−8^	0.8	51 (28)	0	55	0.126
	*rad52 dnl4*	4.3×10^−7^	4.3	31 (0)	0	0	<10^−6^
	*rad52 dnl4 rad1*	7.6×10^−8^	0.8	15 (0)	0	0	<10^−3^
uracil prototrophy (*RPL20A*)	*WT (URA3 overlap of 58 bp)*	0.9×10^−7^	1	all	0	-	-
	*WT (URA3 overlap of 401 bp)*	5.6×10^−6^	62	all	0	-	-
	*pol32 (URA3 overlap of 401 bp)*	2.1×10^−6^	23	all	0	-	-

a results from Koszul *et al.*, 2004.

b the number LTR-mediated SDs is indicated between parenthesis.

nd, not determined.

To confirm this surprisingly high value, an independent estimation of the duplication rate was achieved by designing a new selection assay based on the recovery of uracil prototrophy instead of growth recovery. In this system, *RPL20A* is not deleted and therefore both parental and duplicated strains show the same growth rate. Two truncated copies of the *URA3* gene, overlapping by only 58 bp, were introduced in place of the two Ty3 LTRs, *YORsigma3* and *YORsigma4* located on either side of *RPL20B* and separated by 115 kb (YKFB614, Figure 1B). In the original growth-assay, approximately half of all SDs (48%, [20]), corresponds to an intra-chromosomal 115 kb direct tandem duplication between these two LTRs (Figure 1B). The size of the *URA3* overlapping sequences (58 bp) is comparable to the largest identity region shared by the two LTRs (44 bp). Thus, recovery of a functional *URA3* gene at the duplication breakpoints is indicative of direct tandem *ura3*-mediated SDs, mimicking the 115 kb LTR-mediated SDs. In this system, the duplication rate was evaluated to 0.9×10^−7^ event/cell/division (using the median method [22], Table 1). To further test this rate, we created a *rpl20AΔ* derivative of YKFB614 (YKFB605, Table S1) and examined its duplication rate using the growth recovery assay. We found a rate of 1.7×10^−7^, consistent with the fact that the rate derived from the *URA3* assay represents only half of real duplication rate and close to our present estimate of 1×10^−7^.

This rate only accounts for duplications encompassing the *RPL20B* reporter gene, located on the right arm of chromosome XV. Therefore, extrapolation to the whole genome would lead to a much higher rate, suggesting that spontaneous SD events must be extremely common in yeast populations. For instance, a very high rate of histone gene amplification, compensating for decreased level of histones, was shown to result from recombination events between two Ty1 retroelements leading to supernumerary circular chromosomes [23]. However, our present estimate of SD rate is several orders of magnitude higher than that of other types of chromosomal rearrangements characterized in different studies using native yeast chromosomes [24],[25]. This discrepancy could be explained by the absence of spatial constraints imposed on the boundaries of the SDs in our screen while in the other studies, the location of one end of the rearrangements is restricted within a narrow chromosomal region.

### A Replication-Firing Defect Promotes SD Formation

To investigate the molecular mechanisms involved in SD formation, we used our selection system in conditions where replication is altered. Clb5 is a B-type cyclin known to activate late replication origin: in a *clb5*Δ strain S-phase duration is increased and the replication pattern modified [26],[27],[28]. The rate of SD formation is greatly increased in *clb5Δ* (730x compared to the control strain, Table 1), unveiling the broad genomic instability induced by the perturbation of replication origin firing. Interestingly, the relative proportions of intra- *versus* inter-chromosomal SDs are conserved compared to the wild-type (WT) strain (Table 1). Although this is at the limit of statistical significance, the proportion of the 115 kb LTR-mediated duplications (between the two Ty3 LTRs, *YORWsigma3* and *YORWsigma4*, Figure 1B) is slightly increased in *clb5Δ* (62% compared to 48% in the WT, P = 0.05 Fisher's exact test, Table 1). It is noteworthy that these LTR sequences lie next to tRNA genes whose transcription by PolIII is known to stall the progression of replication forks [29]. The size distribution of the intra-chromosomal SDs in *clb5Δ* remains globally similar to that of the WT (see Figure 1C, in which WT and *clb5Δ* strains have radically different SD distributions as compared to *rad52* and *rad1* mutants).

**Figure 2:**
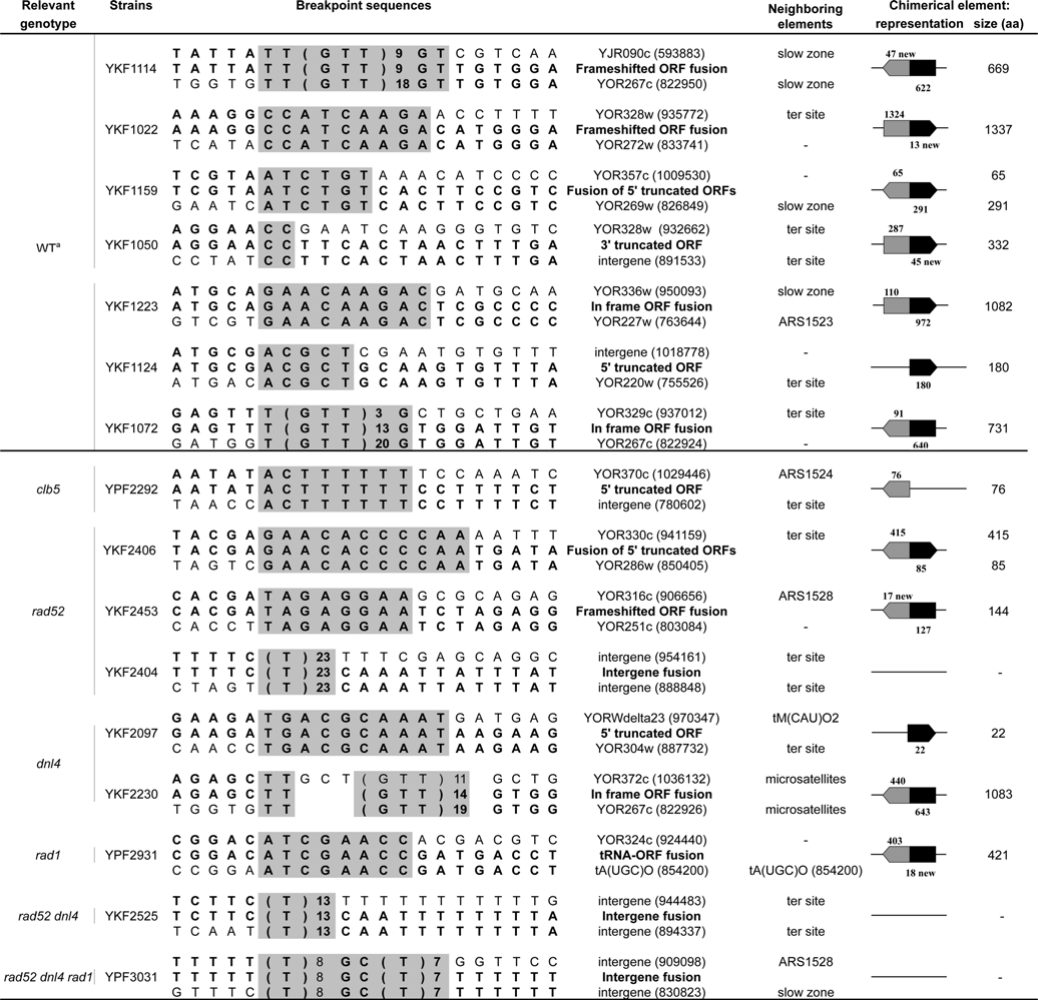
Representative breakpoint sequences of non LTR-mediated duplications. Only events leading to chimerical ORF are presented. ^a^ WT junctions are from [20]. Top and bottom sequences correspond to centromere-distal and -proximal sequences, respectively, followed by the name of the genetic element involved at the junctions. Shaded areas indicate the regions of sequence identity shared by these two sequences and correspond to the breakpoint *per se*. The coordinates in brackets correspond to the first nucleotide position within the shaded areas. For each strain, the middle sequence corresponds to the actual breakpoint sequence followed by a description of the chimerical genetic element recovered at the junction. Neighboring elements correspond to sequences known to participate or interfere with replication, with slow-zone corresponding to inflection point in the replication pattern (i.e. regions where fork progression slows down, [67]), ter site to termination regions, and ARS to autonomous replicating sequences. On the right, the schematic representations with orientated grey and black boxes represent the structure of the chimerical elements generated with the sizes of the corresponding chimerical ORFs (in aa). The contribution (in aa) of each of the two elements involved in the fusion is indicated above and below the corresponding boxes. Amino acids encoded by a frame different from that of the original elements are referred as “new”.

The breakpoint sequence of a non-LTR mediated SD was characterized through comparative genomic hybridization (CGH) and PCR amplification, revealing the presence of microhomologies at the junction (Figure 2). This junction is identical to the one found in the strain YKF1080 strain isolated in our original control screen [20]: the same two copies of a 9 nucleotide microhomologous sequence (ACTTTTTTT) have been involved in the formation of two independent SDs, recovered in two different genetic backgrounds. There are 2367 copies of this sequence in the genome, 47 of which interspersed between the two recombining sequences. It is unlikely that this repetitive use occurred by chance and therefore must be indicative of a chromosomal rearrangement hotspot. Interestingly, the centromere-proximal sequence lies next to an autonomous replicating sequence (ARS1524) and the centromere-distal one corresponds to a replication termination site (Figure 2), which could explain their recurrent use in SD formation (below).

Altogether these results suggest that the mechanisms of SDs formation are similar in WT and *clb5* strains. In addition, the dramatic SD rate increase associated with the *clb5* mutation could be related either directly to the perturbed S-phase origin firing and/or to indirect effects of this perturbation onto replication. In this regard, the reported Rad9-dependent activation of the replication checkpoint key protein Rad53, by late S-phase, strongly suggests that a *CLB5* deletion results in the formation of replication-induced DNA breaks [30]. Such breaks could therefore represent the precursor lesions leading to SDs.

### Broken Replication Forks Are Processed into SDs

In order to test whether broken replication forks could correspond to these precursor lesions, we monitored SD formation in cells treated with camptothecin (CPT), a topoisomerase I inhibitor. CPT stabilizes the covalent intermediate that forms during the catalytic DNA nicking-closing cycle of Top1, and CPT cytotoxicity results from the conversion of single strand nicks into double-stranded DNA ends when a moving replication fork collides with a CPT-Top1 complex [31]. The rate of SD formation is strongly increased in an exponential culture treated for 3 hours with 10 µg/ml of CPT (x 320, Table 1). This observation could be explained if the precursor lesions leading to SDs were indeed double-strand DNA ends that in standard conditions would result from replication accidents. Several other lines of evidence support this hypothesis. First, SD breakpoints often correspond to sequences known to interfere with the replication forks progression (Figure 2 and [20]). Moreover, replication-induced DNA damages in a *clb5Δ* strain [30] would explain the massive increase in SD formation observed in the absence of this cyclin.

These lesions are likely to impede fork progression and trigger the activation of the replication checkpoint. Besides preventing fork collapse and the subsequent formation of DNA breaks, the replication checkpoint also regulates a large variety of cellular events including repression of late-replicating origins, inhibition of mitosis and induction of DNA repair genes [32]. This trans-acting branch of the replication checkpoint relies on the hyperphosphorylation of Rad53 that can be specifically abrogated with a *mrc1^AQ^* allele [33]. To determine whether SDs result primarily from S-phase induced DSBs rather than being secondary byproducts of the checkpoint activation, we characterized SD formation in a *mrc1^AQ^* mutant in presence of hydroxyurea (HU). By inhibiting the ribonucleotide reductase activity, HU slows down replication fork progression and promotes the formation of ssDNA at the forks, which is sufficient to activate the checkpoint in normal cells [32],[34]. In a *mrc1^AQ^* strain and in the presence of HU (100 mM for 3 hours; Methods), the integrity of stalled replication forks is maintained while the trans-acting branch of the replication checkpoint is suppressed. In these conditions we found no significant differences between HU-treated and untreated cultures in either checkpoint competent or deficient cells (2 to 5 fold increase, Table 1). These results demonstrate that neither stalled replication forks nor the Rad53 hyperphosphorylation-mediated functions of the replication checkpoint are sufficient to stimulate SD formation. Altogether, the above findings strongly suggest that broken forks are the precursor lesions that are directly processed into SDs.

### SDs Are Generated through Replication-Based Mechanisms that Require Pol32

Free DNA ends generated at broken forks are thought to be repaired primarily by strand invasion of the sister-chromatid, followed by the assembly of a new fork and subsequent replication up to the chromosome end (or to the next replication fork). This break-induced replication (BIR) mechanism can occur through successive rounds of strand invasion and dissociation, and lead to chromosomal rearrangements if reinvasion occurs within ectopic repeated sequences [35]. We explored a potential role for BIR-related mechanisms by investigating SD formation in a *pol32*Δ strain. Pol32 is a non-essential subunit of *S. cerevisiae* major replicative DNA polymerase Polδ and is required for the replication fork assembly that initiates the BIR reaction [36]. Absence of Pol32 completely abolishes the formation of SDs. No mutant carrying duplications of any type were isolated out of 184 independent *pol32*Δ cultures. Thus, although the true duplication rate cannot be calculated, the occurrence of a single event, out of the 184 cultures, would have lead to a reversion rate of 6.9×10^−9^. Although this value is an overestimate of the true duplication rate, it represents a 14-fold reduction compared to the WT control (<0.07, Table 1). These data reveal the crucial role played by Pol32 in the generation of all types of SDs. Given that Pol32 is not required for repair by gene conversion (GC) events, SDs must therefore result principally from replication-based mechanisms rather than from unequal crossing-overs (UCO) between sister-chromatids. In addition, since only half of SDs contain repeated homologous sequences at their junctions [20], classical BIR mechanism involving Rad52-mediated interactions between large sequences of homology could only account for half of all of the events: the other half might result from a Pol32-dependent replication-based mechanism involving microhomologous or low complexity sequences at the site of strand invasion (see below).

### The Endonuclease Activity of the Rad1-Rad10 Complex Stimulates SD Formation

These two Pol32-dependant replication-based mechanisms must rely on an initial step of ectopic strand invasion. The Rad1/Rad10 complex possesses an endonuclease activity required for the removal of non-homologous tails during GC events [37]. This complex is also essential for the Rad52-independent microhomology mediated end-joining (MMEJ) DNA repair pathway [38] and was shown to promote the production of gross chromosomal rearrangements (GCRs) [39]. A deletion of the *RAD1* gene results in a 5-fold reduction of SD formation (x 0.2 as compared to WT, Table 1), suggesting that the endonuclease activity is required to generate duplications. We also noted a substantial (although not highly significant) decrease in the proportion of LTR-mediated SDs compared to WT (14% vs. 48%, respectively; P = 0,06, Table 1), suggesting that Rad1 is directly involved in the generation of BIR-mediated SDs rather than of microhomology-related ones. Consistently, the proportion of small (<115 kb) intra-SDs is increased and reminiscent of the distribution of rad52-independent duplications (Figure 1C; below).

One SD breakpoint from a *rad1*Δ mutant was sequenced, revealing an eight-nucleotide homology at the junction (Figure 2) and implying that, despite its predominant role in MMEJ, Rad1 is not required for the generation of microhomology-mediated SDs. Similar microhomologies were reported at the junction of GCRs recovered in *rad1*Δ and *rad10*Δ strains [39]. Interestingly the centromere-proximal microhomologous sequence involved in this rearrangement lies within the tRNA (tA (UGC)O; Figure 2) that flanks YORWsigma3 (the LTR recurrently used in the 115 kb intra-chromosomal SDs). Given that tRNAs transcription is able to stall incoming replication forks, these sequences were proposed to exhibit spontaneous fragility and thus promote chromosomal instability [40]. The eight-nucleotide microhomology sequence could therefore represent the recurrent breaking site which initiates the formation of the common 115 kb LTR-mediated SD: in the presence of Rad1, the 3′ flap sequence between this break site and the LTR sequence would be excised so that a BIR-mediated SD could occur.

### HR-Mediated SDs Result from Rad51-Independent BIR

It is generally believed that most SDs must result from non-allelic recombination events between dispersed repeats, but so far no demonstration for the involvement of the homologous recombination (HR) pathway in SD formation has been clearly established. In a *rad52*Δ strain where HR is abolished the class of 115 kb LTR-mediated SDs is completely suppressed (0 out of 71 independent events compared to 23 out of 48 duplications in the WT, P<10^−6^, Table 1). This result clearly demonstrates that this class of SDs results from Rad52-dependent recombination events between interspersed repeats. These duplication events are most likely resulting from a BIR reaction, since they are also dependent on the presence of Pol32 (see above). Furthermore, while *pol32*Δ exhibits a limited reduction in GC efficiency [36], absence of Rad51 restricts both BIR and GC events, although BIR occurs more frequently than GC among the remaining events [41],[42],[43]. In a *rad51*Δ strain, the rate of SD formation is increased (x 7.7, Table 1). This increase suggests that the lesions that were repaired in wild type through gene conversion or allelic BIR are channeled into non-allelic BIR in *rad51* mutants. In addition, the proportion of inter-chromosomal SDs increases up to 32% (10 out of 31 events) as compared to 12% in the WT (6 out of 48, P = 0.02). All types of LTR-mediated SDs are favored in the absence of Rad51 (71% vs. 48% for the control, P = 0.02, Table 1). These findings suggests that Rad51 prevents recombination events between diverged sequences, such as the two LTR repeats *YORWsigma3* and *YORWsigma4* which share only 76% identity over 319 bp (largest identical domain: 44 bp). This is consistent with the fact that Rad51-independent BIR requires shorter identical regions to achieve strand invasion than Rad51-dependent repair (∼30 bp vs. ∼100 bp, respectively [44]). Therefore, it might be that *RAD51* does not simply suppress recombination between diverged sequenced, but normally promotes gene conversion (and allelic BIR) that usually outcompetes ectopic BIR.

Altogether, the above results strongly suggest the following scenario for the formation of the class of 115 kb LTR-mediated SDs: (i) a DNA free end would arose from a broken replication fork in the vicinity of LTR *YORWSigma3* (potentially stalled within the tA (UGC)O tRNA gene), (ii) repair of the DSB occurs through a Rad51-independent strand invasion of the non-allelic LTR sequence, *YORWSigma4,* (iii) followed by a Rad1-dependent 3′ flap removal and (iv) a Pol32-dependent conversion of this strand annealing intermediate into a replication fork generating a large intra-chromosomal SD through BIR (Figure 1D).

### Divergence between Dispersed Repeats Suppresses the Formation of SDs

To further explore the contribution of homologous recombination to SD formation, a system where SDs result principally from HR events was designed. The two LTR sequences, *YORWsigma3* and *YORWsigma4,* were replaced in this strain YKFB608 by two truncated copies of the *URA3* gene, overlapping with a 401 bp region of perfect identity, such that a *URA3*-mediated intra-chromosomal duplication would restore uracil prototrophy (Figure 1B, Table S1). As expected, all growth revertants isolated in an *rpl20A*Δ background resulted from duplication events corresponding to *URA3*-mediated SDs (data not shown). Although the size of the *URA3* overlapping sequences is similar to the size of the LTRs (401 and 319 bp, respectively), the rate of SD formation showed a 56 time increase compared to the original strain with intact LTRs (5.6×10^−6^ vs. 1×10^−7^, respectively) and a 62 time increase compared to a strain carrying only a 58 bp overlap (5.6×10^−6^ vs. 0.9×10^−7^, Table 1). These results confirm that the accumulation of divergence between dispersed repeats suppresses genome rearrangements, while increasing the length of sequence identity between these repeats promotes genomic instability. Indeed, the mismatch repair system can trigger an anti-recombination activity thereby limiting chromosome rearrangements between diverged repeats [45]. In addition, we monitored the effect of the *POL32* deletion in this HR-based assay. In the absence of Pol32, and in the absence of mismatches between repeated sequences, only a 23-fold increase is observed, as compared to the 62-fold increase characterized in the presence of this protein (Table 1). This corresponds to a 2.7-fold decrease (63/23) in the rate of uracil-prototroph formation in *pol32*Δ, a lesser effect that the >14-fold decrease observed in the growth recovery assay (above). It also shows that in the absence of mismatches between repeated sequences, not all SDs require Pol32. These Pol32-independent SDs likely result from UCOs between the repeated identical URA3 sequences. In the original assay, similar UCO events involving the flanking LTRs are probably suppressed due to divergence between the sequences.

### Non HR-Mediated SDs Result from Microhomology/Microsatellite-Induced Replication (MMIR)

The rate of SD formation in a *rad52Δ* strain is slightly higher than in WT (2.8-fold increase, Table 1), revealing that duplications can form even when HR is abolished (as suggested previously in [46], although using a very different system). The SDs recovered in a *rad52Δ* background appear radically different from those obtained in the WT strain. First, there is a significant decrease in the proportion of inter-chromosomal events, since all 71 SDs but one correspond to intra-chromosomal duplications (*versus* 6 out of 48 events in the WT, P = 0.02, Table 1). Second, the size distribution of intra-chromosomal SDs is significantly biased towards smaller segments as most of them (57 out of 71) are smaller than 115 kb (Figure 1C). Third, sequencing of the breakpoints revealed that only microhomologous (between 8 and 9 nt) and low complexity sequences (polyT) are now used to generate SDs (Figure 2). Interestingly, a recent report proposed that the large SDs in the human genome that cause the dysmyelinating PMD disease might result from replication fork stalling followed by homology-independent template switching, relying instead on the presence of microhomologies [47]. Our sequenced breakpoints once again coincide with replication-related elements, such as ARS, termination sites and tRNAs (Figure 2). Given the location and the nature of the initiating lesions, as well as the strict dependency to Pol32 (see above), we conclude that the non-HR mediated SDs result from a new mechanism that would rely on an initial Rad52-independent recombination event, occurring between 5 to 10 bp of microhomology or stretches of low-complexity DNA sequences such as microsatellites, followed by a Pol32-dependent fork assembly initiating DNA synthesis (Figure 1D). Therefore, we propose to designate this new mechanism MMIR for microhomology/microsatellite-induced replication.

### Non Homologous End Joining (NHEJ) Does Not Contribute to SD Formation

All of the above data clearly show that spontaneous SDs result from replication-based mechanisms. Nevertheless, the putative contribution of NHEJ to SD formation was addressed. NHEJ is strictly dependent on the activity of the ATP-dependent DNA ligase, Dnl4 (also named Lig4), as well as that of the Yku70/Yku80 DNA binding complex [48],[49] When *DNL4* is deleted, SDs arise at a slightly lower frequency (x 0.8, Table 1), and present a similar proportion of LTR-mediated events (Table 1). Among the non-LTR mediated events, two junctions were sequenced. One lies next to a microsatellite (GTT)_14_ identical to the one found in the WT strain YKF1057 [20], again corresponding to the recurrent use of a particular sequences at SD boundaries. The other corresponded to a 10 bp-long sequence of microhomology (TGACGCAAAT), repeated 109 times in the genome, in which the two recombining copies lie next to a tRNA gene and a replication termination site (Figure 2). Although all of these characteristics are very similar to SDs generated in the WT strain, there is, however, a significant decrease in inter-chromosomal duplications (0 out of 51 in *dln4Δ versus* 6 out of 48 in WT, P = 0.01, Table 1), suggesting that Dnl4 is required for inter-chromosomal SD formation. However, the junction sequences of the 6 inter-chromosomal events in WT were indicative of either LTR-mediated or microsatellite-mediated events (3 occurrences each, respectively) [20],[21]. These sequences differ strongly from those usually found at NHEJ-mediation junctions (1–4 nucleotides complementary sequences, [50]), suggesting that, in addition to its well-described role in NHEJ, Dnl4 might participate in the replication-based mechanisms of inter-chromosomal SD formation.

### SDs Are Still Being Formed in the Absence of All Known DSB Repair Pathways

In the double mutant *rad52*Δ *dnl4*Δ the rate of SDs formation is moderately increased (x 4.3) compared to WT (Table 1). It is noteworthy that in the GCR assay, developed by Kolodner and collaborators, the concomitant deletion of *RAD52* and *DNL4* completely abolished the formation of non-reciprocal translocations since all GCRs observed resulted from telomere additions [51]. This discrepancy underlines the differential genetic requirements between SD and other GCR mechanisms. Deletion of *RAD1* in the *rad52*Δ *dnl4*Δ strain reduced the SD rate to a level similar to that of the WT (Table 1), as expected since Rad1 promotes SDs formation (above). The type of SDs, the size distribution as well as the breakpoint sequences isolated in the progenies of these double and the triple mutants strains, are similar to the ones characterized in the *rad52* single mutant (Figures 1C, Table 1 and Figure 2). Therefore, when both HR and NHEJ are abolished and when MMEJ is, at least, severely compromised (as in *rad52*Δ *dnl4*Δ *rad1*Δ strain*)*, SDs still occur at a WT rate. Since SDs would mainly result from the replicative-repair of a one-ended DSB generated at a broken fork, the concomitant mutations of the 3 major DSB repair pathways should severely reduce if not abolish SD formation. The maintenance of a rate of formation similar to WT and the physical characteristics of SDs in this background suggest that MMIR could represent a new DSB repair pathway. Alternatively, these SDs could be formed by template switching, in the absence of any DSB, as suggested for the formation of *PLP1*-encompassing SDs in the human genome [47].

### Chimerical Genes Leading to Protein Innovations Are Produced at Microhomology-Mediated Breakpoints

Altogether, 26 SD breakpoints were sequenced (this work and [20],[21]) allowing the identification of 13 different chimerical Open Reading Frames (ORF) containing either microhomologies or trinucleotide repeats at their junctions (Figure 2). Microhomologies were found at breakpoint junctions in *rad52*Δ, *dnl4*Δ and *rad1*Δ backgrounds, where HR, NHEJ and MMEJ are impaired, respectively (Figure 2). This shows that these sequences can be used in the absence of all known DSB repair pathways. Because of their extremely high genomic density, the impact of microhomologies in SD formation, and more generally in genome dynamics, is likely to be important. For instance, the 8 to 10 nucleotide breakpoint sequences characterized in the *rad52*Δ, *dnl4*Δ and *rad1*Δ backgrounds are found in the *S. cerevisiae* genome from 109 times for the less frequent (TGACGCAAAT), and up to 793 times for the most common (TAGAGGA, Figure 2). Chimerical genes arise either from in- or out-of-frame ORF fusions (3 occurrences each), from 3′ or 5′ ORF truncations (1 and 5 occurrences, respectively) or from the fusion between an ORF and a tRNA (Figure 2). These fusions can generate new proteins and thus represent a potential mechanism of protein evolution. Whereas chimerical ORFs resulting from translocation and inversion events are associated with the concomitant lost of the original gene integrity, SD-mediated chimerical genes formation leave intact the original copies of the genes involved at the breakpoint. For instance, in addition to the original full-length gene a truncated copy of *SGS1* (homolog of human BLM) has been found in the pathogen yeast species *Candida glabrata* [52]. This powerful mechanism allows SD-mediated chimerical genes to explore new combinations that might be counter-selected for in the cases of classical translocation- or inversion-mediated events. In-frame ORF fusions (3 cases) might result in new protein architectures by combining previously existing domains. In addition, SD-mediated frameshift fusions and ORF truncations may result in true protein innovations at the junctions by promoting the transcription of otherwise non-coding sequences. The corresponding transcripts would encode entirely new amino acid combinations. For instance, the frameshift chimerical ORF generated in strain YKF1114 comprises a coding sequence whose last 47 amino acids (from the breakpoint to the stop codon) represent a truly new protein segment that shows no similarity to the rest of the yeast proteome. Such peptides were found in 5 out of the 13 chimerical ORFs characterized (Figure 2). Although relatively small (average size of 28 amino acids), these peptides are new genomic features and may generate new protein domains. Despite their known association with diseases and genome rearrangements, it has been proposed that SDs have been fixed in the human genome to increase copy number of fusion genes originating from initial duplications of gene-rich core regions, eventually leading to the emergence of new gene families that are either unique to hominoids or considerably diverged when compared with other mammalian species [53].

## Discussion

Given their close association with various genomic disorders and cancers and their broad evolutionary impact, SDs and CNVs represent one of the most important discoveries that stem from the human genome project. Careful computational characterization of SD breakpoints in the genomes of human and other primates has suggested an important role for *Alu*-mediated recombination in the production of intra- and inter-chromosomal SDs [54]. However, *Alu* elements are found in only 30% of the SD breakpoints and sequences presenting the physicochemical properties of “fragile sites” were shown to play an important role as well [55]. In addition, recent studies have proposed that SDs and other complex rearrangements associated with genomic disorders would result from replication-based mechanisms rather than from more classically invoked recombination-based models such as non-allelic homologous recombination between dispersed repeats [47],[56],^57^. Although essentially based on breakpoint analyses, these studies reach conclusions similar to those drawn here from experimental evidences.

We found a massive SD rate increase both in a *clb5*Δ strain where origin firing is perturbed, S-phase is lengthened and DNA damages are detected by late S-phase [26],[27],[28],[30] and in CPT-treated cultures in which single-strand nicks are converted into broken forks [31]. The recurrent use of genetic elements known to interfere with replication forks progression at SD breakpoints (tRNA, microsatellites, ARS, replication slow zones and termination regions, Figure 2) also points towards the involvement of replication and the use of broken forks as the initiating lesions in the pathways leading to SDs. In addition, the finding that all SD formation requires the nonessential Polδ subunit Pol32 shows that duplications results from replication-based mechanisms rather than from UCOs, which are suppressed by the natural DNA divergence between dispersed repeats such as LTRs. It also suggests that BIR, which also requires Pol32 to initiate new DNA synthesis [36] would be the mechanism by which SDs are formed. However, BIR is a homologous recombination process which implies an initial Rad52-dependent invasion step necessitating large sequences of homology between the recombining molecules (reviewed in [58]). These requirements imply that BIR cannot be the unique pathway leading to SDs, because only half of the SDs are generated through a Rad52-dependent recombination event between homologous sequences (Table 1). The remaining SDs occur independently from both Rad52 and large homologous regions and are generated through recombination between short identical/low complexity sequences. A Rad52-independent half-crossover pathway was previously described [59],[60] and unequal half-crossovers in G2 could also generate tandem duplications. However, the class of Rad52-independent SDs described here involves only microhomology/microsatellite sequences at breakpoints and requires Pol32, two characteristics that are hardly compatible with the half-crossover pathway. Given its unique substrate and genetic requirements, this new mechanism of SD formation has been called microhomology/microsatellite-induced replication, or MMIR, because it brings together characteristics from both MMEJ (*ie.* recombination between microhomologies in a Rad52-independent manner, [38]) and BIR (*ie.* a Pol32-dependent DNA synthesis step, [36]). In addition, we show that MMIR-mediated SDs still form in the absence of all known DSB repair pathways (HR, NHEJ and MMEJ) suggesting that MMIR could represent a new repair pathway. Alternatively, one cannot exclude that MMIR-mediated SDs would arise in the absence of any DSB as a result of template switching events as it has been suggested for the large *PLP1* duplications that cause the dysmyelinating PMD disease in human [47].

Altogether, our results provide the first experimental deciphering of the molecular pathways leading to SDs, demonstrating that two alternative replication-based mechanisms, BIR and MMIR, are responsible for the spontaneous SD formation in the yeast genome (Figure 1D). While these two pathways probably use similar precursor DNA lesions and share the Pol32 requirement, they differ from one another by their recombination substrate and their dependency to HR proteins (Rad52, Rad51 and Rad1). To our surprise, the Dnl4 ligase seems to contribute to the formation of inter-chromosomal SDs resulting from either BIR or MMIR. A similar Dnl4 requirement has been described for the formation of non-reciprocal translocations in *S. cerevisiae* [61]. Dnl4 has a preponderant role in NHEJ and also participates in MMEJ [38],[48],[49]. However, the sequences characterized at inter-chromosomal SD breakpoints (LTRs and microsatellites) are very different from typical signatures of either NHEJ or MMEJ events [38],[50]. These results suggest that the role played by Dnl4 in inter-chromosomal SD formation would be different from the other known functions of this protein.

**Figure 3:**
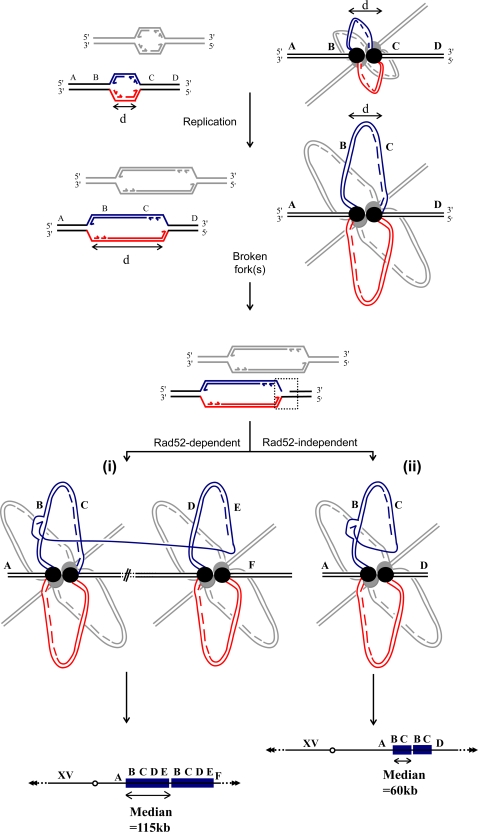
Potential interplay between replication factories and SD formation. The top two drawings illustrate the conservation of the physical distance between two forks within the same replication bubble as elongation proceeds. Red/blue and grey bubbles symbolize replicons located on two different chromosomes but co-localizing within the same replication factory. A broken fork can be repaired either in a Rad52-dependent (i) or in a Rad52-independent (ii) manner. Rad52-dependent annealing could be achieved through interaction with a sequence from either the same or a different replication bubble (symbolized by the sharp sign between the two replication factories) leading to intra- or inter-chromosomal SD. In a *rad52*Δ strain, SDs are on average shorter and almost exclusively intra-chromosomal (bottom right schematic representation), suggesting a Rad52-independent preferential association between sequences originating from the same replication bubble during the annealing step. A schematic representation of the resulting duplications is presented at the bottom.

Discrete microhomology/microsatellite sequences are recurrently used at SD breakpoints although hundreds, even thousands, of other identical copies are dispersed within the genome. These particular regions thus behave as duplication hotspots. Interestingly, they often correspond to genetic elements linked to replication initiation, progression and termination (e.g. ARS, termination regions, tRNAs, replication slow zones; Figure 2). Such correlation suggests that genomic architectural constraints may favor interactions between specific loci, for instance through promoting spatial proximity during replication. In yeast, two replication forks originating from the same replicon co-localize in the nucleus within a replication factory, a spatial location likely to harbor other forks as well [62]. The tight link between replication and SD formation raises interesting questions with regard to the influence of these factories on eukaryotic genome stability (Figure 3). A single broken fork could be repaired either in a Rad52-dependent or -independent manner (Figure 3i or ii, respectively). The invading broken strand would presumably correspond to the lagging strand template where more ssDNA is exposed at the forks [32]. Given that SD formation requires Pol32, the displacement of the lagging strand would also be compatible with the recent finding that lagging strand replication is performed by Polδ [63]. SDs recovered in the absence of Rad52 present a relatively smaller size (median = 60 kb), reminiscent of the size of a replicon bubble in yeast. This may proceed from the possibility for a DNA free-end to interact spontaneously in a Rad52-independent manner with a sequence present in its vicinity within in the same replication factory (Figure 3ii). In contrast, in a WT background where Rad52 is present, homology search would promote strand invasion between more distant sequences possibly located in different replication bubbles/factories, and thus generate larger duplications.

Interestingly, in highly aggressive cases of neuroblastoma, an heterogeneous pediatric cancer, segmental chromosome instability results in unbalanced chromosome translocations, sometimes associated with additional aneuploidies [64]. These genomic profiles are formally similar to the different classes of inter-chromosomal duplications characterized in *S. cerevisiae* [20]. Whereas BIR is the mechanism usually invoked to account for the development of such chromosomal alterations [65], the absence of repeated sequences at the breakpoints of many of these rearrangements suggests that MMIR may be an important path towards development of cancer.

## Material and Methods

### Yeast Strains

All strains are derivatives of *S. cerevisiae* BY4743 (*MAT* **a**/α, *his3*Δ*1/his3*Δ*1, leu2*Δ*/leu2*Δ, *met15*Δ*/MET15, lys2*Δ*/LYS2, ura3*Δ*/ura3*Δ) [66]. Strain names and their corresponding genotypes and origins are summarized in Table S1. Mutations were obtained either directly through a PCR-based deletion strategy or from EUROSCARF strains where the original geneticin resistance cassette KanMX4 was replaced by another resistance cassette. All constructions were verified by PCR and Southern blot analysis. For each mutation monitored, a diploid parental strain heterozygous for both the *YMR242c* (*RPL20A*) deletion and the deletion of the tested gene(s) was constructed then sporulated. Spores from the progeny carrying both the *YMR242c* deletion and the tested deletions were analyzed.

### Genetic Screens and Mutation Rate Calculations

In the growth-recovery assay, duplication rates were calculated from Luria-Delbruck fluctuation tests, either by using the 0 term of the Poisson law (p = 1−e^lnf0/ndiv^) when a small subset of all cultures contained revertant cells (see [20] for details), or using the median method when most of the cultures were overtaken by revertants [22]. In previous studies, the doubling time of a revertant culture was estimated to be twice as fast as the slow growing parental strain [20],[21]. However, in the culture conditions where the selection assay was performed (serial dilutions in 6 ml YPD in 24-wells plates), careful measurements revealed that the time needed for revertant cells to overtake slow growing populations was longer than predicted and was strain dependant: the doubling time of a duplicated strain is actually 1.3 to 1.4 times smaller than that of the slow growing parent, depending on the mutant background. This discrepancy resulted in a strong effect on the duplication rate estimation compared to our former studies (from 2×10^−9^ to 1×10^−7^ per cell per division in control strain).

In the strains used for the uracil-prototrophy recovery assay, the *RPL20A* gene is not deleted (see Table S1) and both parental and duplicated strains show the same growth rate. Two truncated copies of the *URA3* gene, covering either the 5′ or 3′ half of the gene and overlapping by either 58 bp or 401 bp, were introduced in place of *YORWsigma3* and *YORWsigma4* (strains YKFB614 and YKFB608, respectively, Figure 1B). The rate of appearance of uracil autotrophic colonies was determined by a fluctuation test analysis using the median method [22]. Briefly, ten independent YPD cultures, inoculated with ∼200 cells, were grown at 30°C to ∼3×10^8^ cells/ml. Cells were plated on uracil lacking medium, incubated at 30°C for 2 days and [ura+] colonies were counted. The breakpoint junction indicative of a 115 kb *ura3*-mediated direct tandem duplication was sought through PCR amplification of the region. All [ura+] colonies analyzed carried such duplications, resulting from the fusion of the two URA3 overlapping sequences.

### Chemical Treatments

Independent colonies (2×10^7^ cells) from strains YKF120c and YBaG398 were inoculated in 24 wells plates containing 6ml YPD, and cultivated under agitation during 6 generations at 30°C. Approximately 2×10^6^ cells from each well were then inoculated into either fresh YPD medium, YPD supplemented by 100 mM Hydroxyurea (HU, Sigma) or YPD supplemented by 10 µg/ml Camptothecin (CPT, Sigma), and incubated for 3 hours. After wash approximately 2×10^6^ cells from each well were inoculated into fresh YPD medium. Every 10–11 generations, similar aliquots from each well were re-inoculated into fresh YPD medium. Between every cycle, a sample of the culture was plated onto YPD plates at a density of ∼2×10^2^ to 5×10^3^ cells/plate and incubated at 30°C (above; [20]).

### Pulse-Field Gel Electrophoresis, Comparative Genomic Hybridizations, and Sequencing of the Junctions

Electrophoretic karyotypes of parental and revertant strains, as well as genomic DNA extraction and labelling, were performed as described [20]. Labelled DNA was hybridized against either PCR product-based (Ecole Normale Superieure, Paris France and MWG Biotech) or oligo-based yeast whole-genome arrays (Affymetrix, YG-S98). Arrays were analyzed with the GenePix Pro5.0 or with the Affymetrix GeneChip software, respectively. A genomic ratio for each ORF was defined as the ratio between normalized spot intensity of the revertant and parental strains, from which the mean of all spot intensities ratios was subtracted. SD junctions were PCR amplified. Products were purified using gel extraction columns (NucleoSpin, Macherey Nagel) and sequenced by the Genome Express company (Cogenics).

## Supporting Information

Table S1 Strains used in this work.
(0.04 MB PDF)
