# Supplementary material for: Segmental Duplications Arise from Pol32-Dependent Repair of Broken Forks through Two Alternative Replication-Based Mechanisms
Source: PLoS Genet. 2008 Sep 5;4(9):e1000175. doi: 10.1371/journal.pgen.1000175 (PMC2518615; doi:10.1371/journal.pgen.1000175)
Supplement: Table S1 — Strains used in this work. (0.04 MB PDF) [file pgen.1000175.s001.pdf]

Supplementary Table 1		
Strain name	Genotype	References
YKF120c	*MATα, his3D1, leu2D0, ura3D0, lys2D0, ymr242c::KanMX4*	(Koszul et al, 2004)
YBaG398	*MATα, his3, ade2, leu2, ura3, can1, ymr242c::PralphaNatR, mrc1::His5(S.pombe), pRS405-mrc1^{AQ}(LEU2)*	This work
YKF160-D	*MATα, his3D1, leu2D0, ura3D0, lys2D0, ymr242c::KanMX4, clb5::URA3*	This work
YBaG6843	*MATα, his3D1, leu2D0, ura3D0, lys2D0, ymr242c::PralphaNatR, pol32::KanMX4, Dcan1::HygR*	This work
YKF198	*MATα, his3D1, leu2D0, ura3D0, ymr242c::KanMX4, rad52::LEU2*	This work
YKF142-A	*MATα, his3D1, leu2D0, ura3D0, lys2D0, ymr242c::KanMX4, rad51::URA3*	This work
YKFB605	*MATa, his3D1, leu2D0, ymr242c::KanMX4, YORWsigma3::URA3D5'(1-538)::HIS3, YORWsigma4::URA3D3'(595-804)::LEU2*	This work
YKFB614	*MATa, his3D1, leu2D0, YORWsigma3::URA3D5'(1-538)::HIS3, YORWsigma4::URA3D3'(595-804)::LEU2*	This work
YKFB608	*MATα, his3D1, leu2D0, YORWsigma3::URA3D5'(1-195)::HIS3, YORWsigma4::URA3D3'(595-804)::LEU2*	This work
YKFB609	*MATα, his3D1, leu2D0, YORWsigma3::URA3D5'(1-195)::HIS3, YORWsigma4::URA3D3'(595-804)::LEU2, pol32::KanMX4*	This work
YPF8064	*MATα, his3D1, leu2D0, ura3D0, ymr242c::KanMX4, rad1::HphMX*	This work
YKF154-A	*MATα, his3D1, leu2D0, ura3D0, lys2D0, ymr242c::KanMX4, dnl4::LEU2*	This work
YKF1547	*MATα, his3D1, leu2D0, ura3D0, met15D0, ymr242c::KanMX4, dnl4::LEU2, rad52::URA3*	This work
YPF8088	*MATa, his3D1, leu2D0, ura3D0, ymr242c::KanMX4, dnl4::LEU2, rad52::URA3, rad1::HpHMX*	This work
